# Prevalence of loneliness and association with depressive and anxiety symptoms among retirees in Northcentral Nigeria: a cross-sectional study

**DOI:** 10.1186/s12877-020-01561-4

**Published:** 2020-04-23

**Authors:** Chima C. Igbokwe, Veronica J. Ejeh, Olaoluwa S. Agbaje, Prince Ifeanachor Christian Umoke, Cylia N. Iweama, Eyuche L. Ozoemena

**Affiliations:** Department of Human Kinetics and Health Education, Faculty of Education, University of Nigeria, Nsukka, Nigeria

**Keywords:** Loneliness, Depression, Anxiety, Older adults and retirement

## Abstract

**Background:**

Retirees face numerous challenges, including disassociation from persons in their social networks in Nigeria. Perceived social isolation or loneliness could impair the quality of life in old age, and lead to mental disorders. However, it is uncertain whether perceived loneliness has an independent association with depressive and anxiety symptoms and comorbid conditions in Nigerian retirees. Therefore, we aimed at examining the association between perceived loneliness, depressive and anxiety symptoms, including comorbid conditions among retirees in Northcentral Nigeria.

**Methods:**

This community-based cross-sectional study enrolled retirees aged 60 years and above in different pension zones from February 2019 to August 2019. A two-stage sampling procedure was used to select the study participants. Data on perceived loneliness, depressive, and anxiety symptoms were collected using the 8-item University of California, Los Angeles Loneliness Scale (ULS-8), and the DASS 21-depression and anxiety subscales, respectively. We collected information on the demographic characteristics using a well-validated structured questionnaire. Descriptive statistics, binary and multivariable logistic regression were used to examine the independent associations between loneliness, depression, anxiety, and anxious depression. *P*-values below 0.05 were considered statistically significant.

**Results:**

The mean age of participants was 71.3 (± 6.01) years, and 54.4% were men. The prevalence of loneliness, depression, anxiety, and anxious depression was 21.8, 52.0, 27.7, and 20.5%, respectively. Retirees with depression or anxiety symptoms perceived that they were lonelier than those without depression or anxiety. The multivariable logistic regression model showed that female gender (AOR 1.49; 95% CI (1.09, 2.00), having secondary education (AOR 2.24, 95% CI (1.40, 3.57) and having higher education (AOR 3.82, 95%CI (2.37, 6.16) were significantly associated with depression. Also, lonely retirees are 1.19 times (AOR 1.19; 95% CI (0.84, 1.69) more likely to be depressed compared to retirees that are not lonely, and the anxious depressed retirees are 314.58 times (AOR 314.58; 95% CI (508.05, 1941.70) more likely to be depressed than those without anxious depression.

**Conclusion:**

The prevalence of loneliness, depression, anxiety, and anxious depression were relatively high among the older retirees. Female gender and advanced age were significantly associated with perceived loneliness, depression and anxiety.

## Background

Loneliness among the elderly is a risk factor for poor health outcomes such as poor quality of life, reduced cognitive functions, depression, and functional disability [1–3]. Loneliness has been identified in the literature as an offshoot of retirement, and retirement has the potential to interrupt social networks [4] and reduce the feasibility of securing other jobs. Often in developing nations, including Nigeria, older adults who retired from work environments could have work-related chronic diseases. Also, they experience a lack of financial security or good welfare package and may face post-retirement challenges [5], which may include loneliness, depressive, and anxiety symptoms.

In Nigeria, most people retire around the age of 60 or 65 years, and other people after 35 years in active service. However, many employees in the universities, private firms, and self-employed individuals retire beyond the age of 60 or 65 years. Furthermore, the proportion of older adults aged 65 years and above is increasing in Nigeria partly due to improving the standard of living and decline in the crude mortality rate (CMR). The Population Reference Bureau [5] and the National Council on Ageing [6] reported that older adults constitute about 3.1% or 5.9 million of the total population of 191 million. This figure represents an increase of 600,000 during the 5 years 2012–2017.

Nevertheless, many Nigerian older adults find it very difficult to adapt to life after retirement [7] and experience mental health problems [8, 9]. For instance, Gureje et al. [8] reported that the lifetime and 12-month prevalence estimates of major depressive disorder among older adults in the Ibadan Study Ageing (ISA) were 26.2 and 7.1% respectively. Similarly, Ojagbemi, and Gureje [9] reported a loneliness prevalence of 16.7% among older adults. Several reasons have been identified for post-retirement challenges experienced by the elderly in Nigeria. These include the rural-urban migration of family members that often leads to a higher likelihood of loneliness in older people, minimizes family or social networks [10], and the breaking down of traditional family support systems for the elderly [11]. Since retirement is a risk factor for loneliness in older adults [4], many Nigerian retirees may be experiencing high levels of loneliness.

Loneliness has been conceptualized in literature. Loneliness has been defined as ‘a discrepancy between one’s desired and achieved levels of social relations’ [12]. The discrepancy could exist in the quantity or the intimacy of the relationships [13]. Also, loneliness refers to an upsetting feeling that is associated with the perception that one’s social needs are not fulfilled by the quantity or quality of one’s social networks [14, 15].

Experience of loneliness is associated with poor health outcomes. Research evidence has established a link between loneliness and depressive symptoms [16–18]. Additionally, the literature indicates that depressive symptoms are prevalent among older people with adverse health outcomes. For instance, depressive symptoms have been associated with reduced quality of life [19], reduced activity levels, and higher mortality [20]. Older adults experiencing perceived loneliness and depressive symptoms have poor general wellbeing [21] and maybe prone to the risk of suicide [22].

Also, lonely older adults may have a combined experience of depressive and anxiety symptoms [23]. The co-occurrence of depressive and anxiety symptoms is known as anxious depression. Anxious depression refers to a major depressive disorder with high levels of anxiety symptoms based on symptom severity scales [24, 25]. High anxiety levels in depression, according to Fava et al. [26], refer to a ‘common subtype of depression that is associated with more impairment, suicidality and treatment resistance, both in younger and older adults’ [27, 28]. However, there is a shortage of studies on the prevalence of perceived loneliness among older retirees in north-central Nigeria and the association of loneliness with depressive and anxiety symptoms in this group.

Hence, understanding the relationship between perceived loneliness, depression, anxiety, and anxious depression in a sample of retirees would provide valuable insight into the appropriate approaches of intervention for improving quality-of-life among older adults in Nigeria. Additionally, ascertaining association between loneliness and mental disorders in retirees could provide apt information on the form of mental disorder that is more independently and significantly associated with loneliness among retirees. Such information could prompt government agencies to formulate policies that seek to integrate retirees in mainstream developmental projects at the community, state and national levels, promote social support and opportunities for social interactions among older adults. This in turn could lower the risk of depression and anxiety disorders in retirees.

Therefore, the study aimed to investigate whether older retirees experience greater feelings of loneliness, the association between loneliness, depression or anxiety symptoms among Nigerian retirees. After that, we examined whether retirees with depression or anxiety experience greater feelings of loneliness than those without depression or anxiety. Finally, we examined whether retirees with anxious depression experience greater feelings of loneliness than those without anxious depression (non-anxious depression).

## Methods

### Design and sample

The current study was a community-based survey of retirees to determine the prevalence of loneliness and its association with depressive and anxiety symptoms. The study was approved by the Health Research Ethics Committee of the Ministry of Health, Kogi State, Nigeria (Ref #: MOH/PRS/465/V.1/007). One thousand one hundred four retirees were recruited from the communities using information given to the leaders of retirees in the pension zones in Kogi State. Retirees were contacted at the monthly meetings held at the designated pension zones. We recruited the participants from February 2019 to August 2019, using simple random sampling and convenience sampling techniques from three different types of pension zones.

Inclusion criteria included the following: age of 65 years or above, absence of ill health at the time of the study, and voluntary informed consent. Exclusion criteria included reluctance to participate in the study or non-issuance of informed consent and the presence of sickness/illness that prevents participation. We used the Leslie Kish single population proportion formula to calculate the study sample size. We assumed the prevalence of loneliness, depression, and anxiety disorders to be 50% in older adults (i.e., to achieve the largest sample size) with a 5% margin of error, 95% confidence level, and 2.5 design effect. The calculated sample size for the study was 384. Afterwards, the sample size (i.e., 384) was multiplied by 2.5 design effect (384*2.5 = 960) and 15% non-response rate was added to 960 (i.e., 144 + 960). Finally, the study sample size was determined to be 1104.

#### Measures

After obtaining informed verbal consent from the participants, we gave a detailed explanation of the study, well-trained research assistants alongside the principal investigators administered the 8-item University of California, Los Angeles Loneliness Scale (ULS-8), and DASS 21-D and DASS 21-D (i.e., the Depression and anxiety subscales), and we assisted the participants in completing the self-reported questionnaires. Interviews were conducted face-to-face, and each interview lasted, on average, 60 min.

#### Dependent variable

##### Loneliness

We used an eight-item short measure of the University of California, Los Angeles Loneliness Scale (ULS-8) developed by Hays and DiMatteo [29] to measure the level of perceived loneliness. The scale is a derivative of the Revised 20-item UCLA scale developed by [30]. ULS-8 has a high level of validity and reliability [31, 32]. The ULS-8 is unidimensional that covers both the frequency and intensity of feelings of loneliness. An example of an item in the ULS-8 includes: “*How often do you feel that you lack companionship*?”. To mitigate the effects of response bias, the word ‘lonely’ was not included in the ULS-8 [33]. The ULS-8 items are assigned a four-point scale: (1) Never, (2) Rarely, (3) Sometimes, and (4) Always. Higher total scores indicated higher levels of perceived loneliness. A Nigerian study [9] has used the shorter version of the R-UCLA (3-item version) scale developed by Hughes et al. [34] in a community-based study. The ULS-8 total scores range from 8 to 32. Given the fact there is no conventional cut-off point for the ULS-8, we used a cut-off score of 24 to classify participants into lonely and not lonely. The cut-off point was used in a previous study [35]. In this study, the ULS-8 has a Cronbach’s alpha of .76 (See Additional file 4).

#### Independent variables

##### Depression and anxiety

We measured depression and anxiety using the short form DASS-21 depression and anxiety subscales [36]. The DASS-21 is a self-report scale for the simultaneous assessment of depression, anxiety, and stress [37, 38]. Research has shown that the DASS-21 scale is primarily sensitive in discriminating anxiety from depression [39]. Also, the DASS-21 has been used in older adults’ population [40, 41]. The DASS 21 has three subscales that measure depression, anxiety, and stress. Each subscale comprises seven questions with Likert response scale ranging from 0 (Did not apply to me at all) to 3 (Applied to me very much or most of the time). The DASS-21 items inquire about depressive symptoms (e.g., feeling downhearted and blue), anxiety symptoms (e.g., feeling close to panic), and general stress symptoms (e.g., having a tendency to over-react to situations). However, in the present study, only the scores for the depression (DASS 21-D) and anxiety (DASS 21-A) subscales were calculated by summing the scores for the relevant items of each sub-case then multiplied by two, following the DASS Scale manual [42]. The DASS 21-D classifies the scores as normal (0–7), mild (8–9), moderate (10–14), severe (15–19) and extremely severe (20 and above) while the DASS 21-A classifies scores as normal (0–9), mild (10–13), moderate (14–20), severe (21–27) and extremely severe (≥ 28) [43]. Higher scores indicated a higher level of severity in each dimension. However, for the screening of retirees with depressive and anxiety symptoms, we used the cut-point of ≥21 for depression and anxiety [43]. Furthermore, it is essential to emphasize the fact that the DASS-21 depression and anxiety scales are valuable screening measures rather than diagnostic tools; thus, the prevalence of depression and anxiety may be underestimated or overestimated. Nevertheless, the discrepancies between the “diagnosed” prevalence and the “screened” rates may not be significant because of the high sensitivity and specificity of the DASS-21 subscales [39]. The Cronbach alphas for the two subscales scale, DASS 21-D, and DASS 21-A were 0.95, and 0.81, respectively (See Additional files 1 & 2). The overall Cronbach alpha for the two subscales was 0.92 (See additional file 3). We used the iterative back-translation technique to subject all the instruments used in this study to cultural adaptation and translation into the local Igala and Igbira languages. This process was based on the translation and back-translation model [44].

##### Covariates

The demographic attributes of the participants were collected via demographic profile form. Demographic variables included age (stratified into 3 groups: 65 to 69 years, 70 to 74 years, and above 75 years old), gender (male and female), marital status (married/single/divorced/widowed), level of education (3 groups: primary education, secondary/post-primary education, and tertiary education), monthly pension earning (4 groups: < #10,000; #10,000 – #19,000; #20,000 - 29,000; > #30,000). In addition, we categorized place of residence as rural or urban according to the Nigerian census categorization.

### Statistical analysis

The sample characteristics were analyzed using descriptive statistics. Independent t-test, *F*-test (one-way ANOVA), and Chi-square test were conducted to examine the differences in loneliness status (lonely or not lonely) with or without depression or anxiety across the participants. We reported means and standard deviations of scores, along with the *t*-values, *F*-values, Pearson’s Chi-square values, degrees of freedom, and the *p*-values. We created two categories for the primary outcomes using the cut-off points, as recommended by Wang et al. [35] and Lovibond and Lovibond [42] for the bivariate and multivariate analyses. Using the cut-point of 24 on the ULS-8 scale, we classified participants into *lonely* (coded as 1) and *not lonely* (coded as 0). We used the cut-off point of ≥21 on the DASS 21-D to categorize participants into *depressed* (coded as 1) and *non-depressed* (DASS 21-D score < 21, coded as 0). Similarly, participants with scores greater than 21 (≥ 21 coded as 1) on the DASS 21-A scale were classified as anxious while those with scores less than 21 (< 21, coded as 0) were classified as non-anxious. Participants that met the criteria for both depression (DASS 21-D ≥ 21) and anxiety (DASS 21-A ≥ 21) were categorized as *anxious depressed* (coded as 1) while those who did not fulfill the criteria were classified as *non-anxious depressed* (coded as 0). After the descriptive analysis of the study variables, we performed the bivariate analysis using logistic regression to identify associations between the independent variable (IV: loneliness), dependent variables (DVs: depression, anxiety, and anxious depression), and covariates (age, gender, level of education, place of residence, marital status and monthly income/pension earning). Subsequently, we included variables with *p* ≤ 0.20 from the bivariate logistic regression analysis into the multivariable logistic regression. All probability tests were two-sided, and all the confidence intervals reported are adjusted for design effects. All analyses were adjusted for socio-demographic variables, depression, and anxiety severity. Before data analysis, we checked for multicollinearity among the variables for all the models. The variance inflation factor (VIF) was less than 10 for the independent variables, showing that there was no problem with multicollinearity (See additional file 5). The odds ratio (ORs) with a 95% confidence interval (CIs) was used to measure the strength of the association. For the final model, Hosmer-Lemeshow goodness of fit statistic (*p*-value > 0.05) was considered a well-fitting logistic regression model [43] (See additional file 6). A *p*-value < .05 was considered statistically significant. All analyses were conducted using IBM Statistical Package for Social Science (SPSS) version 25.0 for Windows (IBM, Armonk, NY, USA).

## Results

### Descriptive statistics

Out of 1104 retirees, 1099 (99.6%) participated in this study. Five participants were excluded from the analysis due to incomplete or missing information. The participants’ demographic characteristics were summarized in Table 1. The mean age of participants was 71.3 (± 6.01) years, and 54.4% were men. About half (47.0%) of the participants were aged 70–74 years, more than half (57.1%) live in the urban area, about half (47.6%) of the participants had tertiary education and more than half (53.8%) were married. The mean scores for loneliness, depression, and anxiety were below their respective scale cut-off points, signifying low levels of loneliness, depression, and anxiety among retirees. The bivariate analysis (Table 2) showed that loneliness was not significantly associated with depressive symptoms (*r* = .02, *p* = .43), anxious depression (*r* = .01, *p* = .68), and inversely related to anxiety symptoms (*r* = −.01, *p* = .74). However, it is quite interesting to note that depression was strongly associated with anxiety among the participants (*r* = .58, *p* < .001). Furthermore, the prevalence of loneliness, depression, anxiety, and anxious depression were 21.8, 52.0, 27.7, and 20.5%, respectively (Table 3). There was a significant difference in the loneliness scores [*t* (1097) = − 1.81, *p* = .049] between the male and female participants. Besides, there was a significant difference in the loneliness status-lonely vs. not lonely, **[***χ*^**2**^ (1) = 7.428, *p* = 0.006] between male and female participants (Table 4).

**Table 1:** Demographic characteristics of the participants

Characteristics	(*n*), M (SD), range	
Age (years)	(1099) 71.3 (6.01), 65–89	
Age group	*n*	(%)
65–69 Years	254	(23.1)
70–74 years	516	(47.0)
75 years & Above	329	(29.9)
Gender		
Male	598	(54.4)
Female	501	(45.6)
Place of residence		
Urban	627	(57.1)
Rural	472	(42.9)
Level of education		
Primary education	206	(18.7)
Secondary education	380	(34.6)
Tertiary education	513	(46.7)
Marital Status		
Single	178	(16.2)
Divorced/Separated	226	(20.6)
Married	591	(53.8)
Widowed	104	(9.5)
Monthly Income (pension earning)		
< #10,000	402	(36.6)
#10,000-#19,000	514	(46.8)
#20,000 - #29,000	121	(11.0)
#30,000 & above	62	(5.6)

**Table 2:** Descriptive statistics and correlations of the study variables

Variables	Mean (SD)	Possible range	Exact range	1.	2.	3.	4.
Loneliness	20.31 (3.59)	8–32	12–30	–	.024	−.010	.013
Depressive symptoms	20.32 (12.62)	0–42	0–42		–	.58**	.429**
Anxiety symptoms	14.02 (10.60)	0–42	0–42			–	.651**
Anxious depression		21–42	21–42				–

*Note*. ** *p* <  0.001

**Table 3:** Prevalence of loneliness, depression and anxiety

Outcomes	*n* (%)
Loneliness	
Not Lonely	859 (78.2)
Lonely	240 (21.8)
Depression	
Non-Depressed	528 (48.0)
Depressed	571 (52.0)
Anxiety	
Non-anxious	795 (72.3)
Anxious	304 (27.7)
*Anxious depression	
Anxious depressed	225 (20.5)
Non-anxious depressed	874 (79.5)

*Note.* Loneliness, ULS-8 score ≥ 24; Depression, DASS 21-D score ≥ 21; Anxiety, DASS 21-A score ≥ 21; *DASS 21-D score ≥ 21+ DASS 21-A score ≥ 21

**Table 4:** Summary of participants’ scores on loneliness, depression, anxiety, and anxious depression in relation to demographic factors

Variable	Total sample (*N* = 1099)		Loneliness				
			ULS-8 scores, *M* (SD)	Lonely (*n* = 240)	Not lonely (*n* = 859)	*t* (df), *χ*^**2**^ (df), *F* (df)	
Age group	*n*	(%)				*F* (2,1096) = 0.681	
65–69 Years	254	(23.1)	20.32 (3.65)	56 (22.0)	198 (78.0)	*χ*^**2**^ (2) = 3.960	
70–74 years	516	(47.0)	20.42 (3.72)	124 (24.0)	392 (76.0)		
75 years & Above	329	(29.9)	20.12 (3.32)	60 (18.2)	269 (81.8)		
Gender						*t* (1097) = −1.813	
Male	598	(54.4)	20.13 (3.50)	112 (18.7)	486 (81.3)	*χ*^**2**^ (1) = 7.428	
Female	501	(45.6)	20.52 (3.68)	128 (25.5)	373 (74.5)		
Place of residence						*t* (1097) = − 1.108	
Urban	627	(57.1)	20.20 (3.64)	127 (20.3)	500 (79.7)	*χ*^**2**^ (1) = 2.143	
Rural	472	(42.9)	20.45 (3.52)	113 (23.9)	359 (76.1)		
Level of education						*F* (2,1096) = 0.053	
Primary education	206	(18.7)	20.35 (3.47)	46 (22.3)	160 (77.7)	*χ*^**2**^ (2) = 0.211	
Secondary education	380	(34.6)	20.34 (3.49)	80 (21.1)	300 (78.9)		
Tertiary education	513	(46.7)	20.27 (3.71)	114 (22.1)	399 (77.8)		
Marital Status						*F* (3,1095) = 1.407	
Single	178	(16.2)	20.05 (3.78)	36 (20.2)	142 (79.8)	*χ*^**2**^ (3) = 3.103	
Divorced/Separated	226	(20.6)	20.58 (3.50)	59 (26.1)	167 (73.9)		
Married	591	(53.8)	20.36 (3.53)	124 (21.0)	467 (79.0)		
Widowed	104	(9.5)	19.83 (3.76)	21 (20.2)	83 (79.8)		
Monthly Income (pension earning)						*F* (3,1095) = 0.997	
< #10,000	402	(36.6)	20.29 (3.62)	94 (23.4)	308 (76.6)	*χ*^**2**^ (3) = 1.240	
#10,000-#19,000	514	(46.8)	20.32 (3.58)	109 (21.2)	405 (78.8)		
#20,000 - #29,000	121	(11.0)	20.65 (3.50)	23 (19.0)	98 (81.0)		
#30,000 & above	62	(5.6)	19.68 (3.67)	14 (22.2)	48 (77.8)		
Depression, *M (SD)*						*t* (1097) = −0.755	
Depressed	571	(52.0)	20.22 (3.47)	134 (23.5)	437 (76.5)	*χ*^**2**^ (1) = 1.849	
Non-depressed	528	(48.0)	20.39 (3.69)	106 (20.1)	422 (79.9)		
Anxiety, *M (SD)*						*t* (1097) = −1.066	
Anxious	304	(27.7)	20.24 (3.57)	73 (24.0)	231 (76.0)	*χ*^**2**^ (1) = 1.165	
Non-anxious	795	(72.3)	20.49 (3.65)	167 (21.0)	628 (79.0)		
Anxious depression, *M(SD)*						*t* (1097) = − 0.416	
Anxious depressed	225	(20.5)	20.28 (3.57)	186 (21.3)	688 (78.7)	*χ*^**2**^ (1) = 0.775	
Non-anxious depressed	874	(79.5)	20.40 (3.73)	54 (24.0)	171 (76.0)		

***Note***. *Depressed, DASS 21-D scores ≥ 21; Anxious, DASS 21-A scores ≥ 21*

### Associations between loneliness, depression, anxiety and anxious depression

Table 5 presents the results of the analyses to examine association between loneliness, depression, anxiety, and anxious depression. We entered depression into the bivariate and multivariable logistic regression models as a dependent variable. In the bivariate analysis, being aged ≥75 years, female gender, and having a tertiary education were associated with depression. Furthermore, loneliness, anxiety, and anxious depression were independently and significantly associated with depression among the participants.

**Table 5:** Binary and multivariable logistic regression of associations between depression, loneliness, anxiety, and anxious depression and demographic factors of participants

	Depression Total sample (*n* = 1099)		Model 1		Model 2	
Variables	Depressed *n* (%)	Non-depressed *n* (%)	COR (95% CI)	*P*-value	AOR (95% CI)	*P*-value
Age						
60–69 Years	136 (23.8)	118 (22.3)	1.00		1.00	
70–74 years	292 (51.1)	224 (42.4)	1.13 (0.84,1.53)	0.424	1.18 (0.82, 1.71)	0.369
≥ 75 years	143 (25.1)	186 (35.2)	0.67 (0.48, 0.93)	0.016	0.73 (0.49, 1.10)	0.131
Gender						
Male	281 (49.2)	317 (60.0)	1.00		1.00	
Female	290 (50.8)	211 (40.0)	1.55 (1.22, 1.97)	< 0.000	1.49 (1.09, 2.00)	0.011
Residence						
Urban	339 (59.4)	288 (54.5)	1.00 (ref)		1.00	
Rural	232 (40.6)	240 (45.5)	0.82 (0.46, 0.73)	0.107	1.01 (0.74, 1.40)	0.941
Education level						
Pry education	81 (14.2)	125 (23.7)	1.00		1.00	
Sec. education	176 (30.8)	204 (38.6)	1.33 (0.94, 1.88)	0.104	2.24 (1.40, 3.57)	0.001
Tertiary education	314 (55.0)	199 (37.7)	2.44 (1.75, 3.39)	0.000	3.82 (2.37, 6.16)	< 0.000
Marital Status						
Single	97 (17.0)	81 (15.3)	1.00		1.00^a^	
Div./Separated	109 (19.1)	117 (22.2)	0.78 (0.53, 1.15)	0.211	–	–
Married	298 (52.2)	293 (55.5)	0.85 (0.61, 1.19)	0.341	–	–
Widowed	67 (11.7)	37 (7.0)	1.51 (0.57, 1.08)	0.104	–	–
Monthly Income (pension or earnings)						
< #10,000	203 (35.6)	199 (37.7)	1.00		1.00^b^	
#10,000-#19,000	261 (45.7)	252 (47.7)	1.02 (0.78, 1.32)	0.909	–	–
#20,000 - #29,000	68 (11.9)	53 (10.0)	1.26 (0.84, 1.89)	0.272	–	–
#30,000 & above	39 (6.8)	24 (4.5)	1.59 (0.92, 2.75)	0.094	–	–
Loneliness						
Not lonely	437 (76.5)	422 (79.9)	1.00		1.00	
Lonely	134 (23.5)	106 (20.1)	1.24 (0.89, 1.74)	0.027	1.19 (0.84, 1.69)	0.036
Anxiety						
Non-anxious	345 (60.4)	450 (85.2)	1.00		1.00	
Anxious	226 (39.6)	78 (14.8)	0.05 (0.02, 0.16)	< 0.000	0.06 (0.017, 0.176)	< 0.000
Anxious depression						
Non-anxious depressed	348 (60.9)	526 (99.6)	1.00		1.00	
Anxious depressed	223 (39.1)	2 (0.4)	0.03 (0.01, 0.02)	< 0.000	314.58 (508.05, 1941.70)	< 0.000

***Note***. *Depressed, DASS 21-D score ≥ 21; Anxious, DASS 21-A score ≥ 21; Anxious depressed, DASS 21-D score ≥ 21 + DASS 21-A score ≥ 21;1.00 indicates the reference variables; COR Crude Odd Ratio; CI Confidence Interval; Sec. Secondary; Pry Primary; Div Divorced; Model 1: unadjusted bivariate associations between depression and loneliness, anxiety, anxious depression and demographic factors of participants; Model 2: associations between depression and loneliness, anxiety, anxious depression adjusting for participants’ demographic variables. In model 2, only variables with p ≤ 0.20 were entered into the multivariable logistic regression; Marital status*^*a*^*and monthly income*^*b*^*were not included in Model 2*

** p < 0.001; * p < 0.05

In the multivariable logistic regression model, female gender (AOR 1.49; 95% CI (1.09, 2.00), having secondary education (AOR 2.24, 95%CI (1.40, 3.57) and having higher education (AOR 3.82, 95%CI (2.37, 6.16) were significantly associated with depression. Also, lonely retirees are 1.19 times (AOR 1.19; 95% CI (0.84, 1.69) more likely to be depressed compared to retirees that are not lonely and the anxious depressed retirees are 314.58 times (AOR 314.58; 95% CI (508.05, 1941.70) more likely to be depressed than those without anxious depression. However, the odds of developing depression were .06 times less likely among anxious retirees than those without anxiety (Table 5).

## Discussion

Using data from a community-based cross-sectional survey of older retirees in Kogi State, North Central Nigeria, the current study aimed to examine the prevalence and associations between loneliness and depression, anxiety, anxious depression, and some demographic factors. In total, 21.8% of retirees reported that they were lonely. Also, more than half (52.0%) of the participants reported having depressive symptoms, while 27.7 and 20.5% reported having anxiety symptoms and anxious depression, respectively. The findings are consistent with prior research that reported prevalence of loneliness, depression, and anxiety among older adults [1, 9, 10, 45–47]. The findings imply that Nigerian retirees experience higher levels of loneliness compared to those reported in previous Nigerian studies [9, 10]. Thus, they are susceptible to depressive and anxiety symptoms and comorbid conditions. This is consistent with previous research that suggests loneliness always co-occur with depression. Although our findings show no significant difference in the loneliness experience of retirees with depression or anxiety and those without, nevertheless, a higher proportion of retirees with depressive or anxiety symptoms experienced loneliness compared to those without the conditions (details are in Table 4). This finding is consistent with prior studies [47, 48].

Also, our findings indicated that male and female retirees differed significantly in their loneliness experiences. Female retirees had a higher mean loneliness score than their male counterparts. Although previous research has reported mixed results on the nexus between loneliness and gender [49], however, the majority of the findings reported that women have higher levels of loneliness than men. Thus, our finding is consistent with the existing literature [50, 51]. A higher prevalence of widowhood has been identified for higher levels of loneliness in women [52]. Consequently, family members, community health workers, and social workers need to provide emotional support and psychosocial interventions for the widowed older adults, especially women, to alleviate their loneliness experience.

Our findings in the bivariate analysis showed that female gender, advanced age (≥ 75 years), having secondary and tertiary education were significantly associated with depression. The findings are consistent with previous studies that reported associations between age [41, 50], low/poor education [53], income [54] and mental disorders such as depression, anxiety and loneliness [41, 50, 53]. For instance, previous studies indicated that women are more likely to experience loneliness, depressive and anxiety symptoms compared to men due to the prevalence of widowhood in women [50–52]. Thus, our findings indicate that psychosocial interventions to reduce mental disorders (i.e., loneliness, depression, anxiety and anxious depression) should specifically target the older women, the middle-old and oldest-old in Nigeria. Such interventions could include the provision of financial incentives, home or community-based regular mental health screening and integration of retirees especially older women middle-old and the oldest-old retirees in social or religious activities that promote their mental health.

One of the aims of this study was to identify independent association between loneliness and depression and anxiety among retirees. In the multivariable logistic regression model, the findings show that loneliness and anxious depression were significantly associated with depression in our sample. The finding coheres with previous studies that reported associations between loneliness, depression, and anxious depression [1, 9, 16–18, 47, 55]. For instance, a systematic review [56] reported association between a high level of loneliness and severe depressive symptoms. The results could suggest the collapse of family or social networks and the traditional family support systems for the elderly in Nigerian communities especially in the rural areas [7]. It is therefore feasible that associations between perceived loneliness, depression and anxious depression among retirees could be due to ageism, truncation of social ties, migration of caregivers (i.e., younger family members) to urban centres in search of elusive job opportunities [7]. Also, the harsh economic conditions in the country could make caring for the elderly by family members or relatives a huge financial burden. Hence, interventions based on social integration; mobilization of social resources to support the elderly; strengthening the fragile family and community support network for older women; and eradication of harmful widowhood practices targeted at older women that predispose to them severe levels of loneliness could significantly reduce mental disorders among the retirees [57, 58]. Also, formal support that mitigates loneliness and that improves social contacts and increase activity levels [55] among older adults should be provided via community health officers (CHOs) and community health extensions workers (CHEW). The intervention should be fully funded by the government and non-governmental organizations (NGOs).

## Strengths and limitations

To the best of our knowledge, only two previous studies [9, 10] have examined loneliness in older adults in Nigeria. Furthermore, only one study [9] had examined the association between loneliness and major depressive order among older adults. None of these studies examined the association of loneliness with anxiety symptoms and anxious depression in retirees in Nigeria. Also, we measured loneliness, depression, anxiety, and anxious depression using well-validated scales. One of the limitations of this study is that we measured loneliness as a single variable (i.e., unidimensional). We did not examine loneliness as a bi-dimensional construct, as suggested in a previous study [59].

Nonetheless, our study has provided valuable evidence on the prevalence of loneliness among Nigerian retirees and its association with depressive and anxiety symptoms. Future studies should examine loneliness as a bi-dimensional construct among Nigerian older adults to further facilitate interventions to reduce loneliness. We used convenience sampling; our study sample could be subjected to selection bias. Nevertheless, we believed this would not have much impact on the generalization of our findings. The retirees’ self-report of loneliness, depression, and anxiety may be undermined by recall bias-such as underreporting or overreporting. Nevertheless, we firmly believe that retirees’ recall of feelings of loneliness, depressive, and anxiety symptoms in the past year might be a significant event. Also, the use of ULS-8, DASS 21-D, and DASS 21-A as screening tools in identifying retirees with the primary outcomes may not be precise. Thus, many diagnoses may have been missed. This could be a limitation as several symptoms of depression and anxiety overlay, and the disorders are usually co-morbid [54]. Therefore, future studies should make use of a clinical interview schedule based on the DSM-V criteria for assessing depressive, anxiety, and mood disorders. Also, this is a cross-sectional study; thus, the causal direction of the relationship between loneliness, depression, and anxiety could be argued to be reciprocal. Longitudinal studies are needed to provide insights into the causal relationship between loneliness and depressive and anxiety symptoms among older adults, including retirees in Nigeria.

## Conclusion

Our findings show the association between loneliness and depressive and anxiety symptoms and their comorbid conditions in older retirees. Depression was related to loneliness, anxiety and anxious depression in our study. Nevertheless, compared to previous studies in Nigeria [8–10], our findings show a higher prevalence of loneliness, depression, anxiety, and anxious depression among retirees. Among the covariates investigated, female gender, advanced age (≥ 75 years), having secondary and tertiary education were associated with loneliness, depression and anxious depression. Therefore, interventions aiming at preventing loneliness, depression and anxiety among older adults could mitigate feelings of loneliness or depressive and anxiety symptoms and anxious depression.

## Supplementary information

**Additional file 1.** Results of reliability test for DASS 21-D scale via Cronbach alpha.

**Additional file 2.** Results of reliability test for DASS 21-A scale via Cronbach alpha.

**Additional file 3.** Results of reliability test for depression and anxiety subscales.

**Additional file 4.** Results of reliability test for ULS-8 via Cronbach alpha.

**Additional file 5.** Outcome of the multicollinearity test for the model.

**Additional file 6.** Results of the multivariable logistic regression model.

## Data Availability

The datasets used and/or analyzed during the current study are available from the corresponding author on reasonable request.

## Abbreviations

ULS-8: The 8-item University of California, Los Angeles Loneliness Scale
DASS 21: Depression, anxiety, and stress scale version 21
VIF: Variance inflation factor
ISA: Ibadan Study Ageing
